# Rapid analysis of composition and reactivity in cellulosic biomass feedstocks with near-infrared spectroscopy

**DOI:** 10.1186/s13068-015-0222-2

**Published:** 2015-03-12

**Authors:** Courtney E Payne, Edward J Wolfrum

**Affiliations:** National Bioenergy Center, National Renewable Energy Laboratory, 15013 Denver West Parkway, Golden, CO 80401 USA

**Keywords:** FT-NIR, NIR spectroscopy, Biomass conversion, Pretreatment, Enzymatic hydrolysis, High-throughput assay, Compositional analysis, Cellulosic biomass, Herbaceous feedstocks, PLS, Reactivity, Biofuels, Multivariate analysis

## Abstract

**Background:**

Obtaining accurate chemical composition and reactivity (measures of carbohydrate release and yield) information for biomass feedstocks in a timely manner is necessary for the commercialization of biofuels. Our objective was to use near-infrared (NIR) spectroscopy and partial least squares (PLS) multivariate analysis to develop calibration models to predict the feedstock composition and the release and yield of soluble carbohydrates generated by a bench-scale dilute acid pretreatment and enzymatic hydrolysis assay. Major feedstocks included in the calibration models are corn stover, sorghum, switchgrass, perennial cool season grasses, rice straw, and miscanthus.

**Results:**

We present individual model statistics to demonstrate model performance and validation samples to more accurately measure predictive quality of the models. The PLS-2 model for composition predicts glucan, xylan, lignin, and ash (wt%) with uncertainties similar to primary measurement methods. A PLS-2 model was developed to predict glucose and xylose release following pretreatment and enzymatic hydrolysis. An additional PLS-2 model was developed to predict glucan and xylan yield. PLS-1 models were developed to predict the sum of glucose/glucan and xylose/xylan for release and yield (grams per gram). The release and yield models have higher uncertainties than the primary methods used to develop the models.

**Conclusion:**

It is possible to build effective multispecies feedstock models for composition, as well as carbohydrate release and yield. The model for composition is useful for predicting glucan, xylan, lignin, and ash with good uncertainties. The release and yield models have higher uncertainties; however, these models are useful for rapidly screening sample populations to identify unusual samples.

**Electronic supplementary material:**

The online version of this article (doi:10.1186/s13068-015-0222-2) contains supplementary material, which is available to authorized users.

## Background

High-throughput methods for the determination of biomass composition and recalcitrance, as it relates to the production of biofuels and chemicals, are increasingly valuable for screening large numbers of plants for suitability as biofuel feedstocks, as well as determining plants that may require further genetic modification of traits that lead to higher fuel yields [1,2]. These methods are vital in reducing the cost of biofuel production by allowing for a more rapid assessment of cost-effective paths forward [1]. The technique of relating near-infrared (NIR) spectral data to a variety of qualitative and quantitative parameters using multivariate analysis has seen a wide variety of applications [3]. In the biofuel sector, NIR rapid analysis has been used at several points in the conversion process, and analysts have developed and published multivariate models to predict composition of native biomass and washed and dried dilute acid pretreated biomass, and dilute acid pretreated biomass slurries [4-6]. NIR spectroscopy has the advantage of requiring little or no sample preparation, is nondestructive, fast, portable, and has process applications. Nonetheless, it is a secondary method and requires primary methods, such as bench top compositional analysis, to build the predictive models for rapid analysis.

The limits of current bench top methods of biomass analysis have been thoroughly discussed in the literature and largely include time and cost as the major limiting variables [1]. Improvements have been made to increase the batch size for these methods, such as with 96-well plates or small vials [7-9]. Nonetheless, these methods are important as the foundation for more rapid secondary methods such as NIR paired with multivariate analysis. Proper execution of these primary methods is also vital because they dictate the quality and performance of the model. Building predictive models is not a trivial process, whether it is in their development or maintenance [10,11]. However, once a model has been established, having used primary methods for quantification of chemical composition and recalcitrance, NIR is a fast and nondestructive method of sample analysis.

Models have been developed for feedstock composition, pretreatment, and enzymatic digestibility using a single feedstock type such as miscanthus, switchgrass, or wheat straw [12-15]. Hames et al. have filed patents to cover a broad range of methods and individual feedstock types [16]. Single feedstock models, though effective, are limiting and unresponsive to the needs of disparate geographical regions cultivating different or multiple energy crops. Second generation biofuels composed of multi-resource/multispecies feedstocks, such as biomass in the Conservation Reserve Program (CRP), will require multispecies broad-based calibrations for rapid analysis. For example, one particular publication of CRP research evaluated 34 sites in the northeastern part of the United States which were determined to have 12 to 60 different species per site with an average of 34 species per site [17]. Multispecies broad-based calibrations can also be useful in situations where a well developed single feedstock population is not available. Here the term, “broad-based” is used to refer to multiple cultivars or varietals within a single species. For example, samples of the feedstock species rice straw may be composed of the main varietals Indica and Japonica and additional varietals within those two groups, such as the varietals Nipponbare, Moroberekan, and Azucena within Japonica.

Multispecies broad-based models have been developed for various compositional constituents; however, there are none that we are aware of that developed multispecies models for composition, sugar release, and enzymatic hydrolysis [18-24]. The forage industry has considered mixed species samples in grasslands. However, the calibrations were developed to predict forage nutritive parameters such as crude protein (CP), neutral detergent fiber (NDF), and acid detergent fiber (ADF) for composition, and various *in vitro* organic matter digestibility assays for carbohydrate convertibility [17,25]. More complex chemical information is required for appropriate evaluation and improvement of biomass conversion processes [2,26]. For biomass feedstock composition, glucan, xylan, and lignin, as the three most abundant cell wall components, are important for composition model development [27]. Glucan and xylan provide information on the major carbohydrates available for bioconversion, while lignin has been implicated in hindering access to carbohydrates, often referred to as recalcitrance [28-30]. Ash is often included because it is relatively easy to measure and has implications on the pretreatment process.

To shed light on the true accessibility of these major carbohydrates for bioconversion, models for release of glucan and xylan through various pretreatment and enzymatic hydrolysis assays are also important. For biochemical conversion of lignocellulosic biomass to fuels, a pretreatment step is regularly used to reduce recalcitrance, thus making complex structural carbohydrates more readily available for hydrolysis. Enzymatic hydrolysis is then often employed to reduce polysaccharides to monosaccharides for further bioconversion. Therefore, while composition provides the carbohydrate content for a given feedstock, it does not necessarily reflect the ability to access these carbohydrates with current pretreatment and saccharification processes.

The primary object of this work was to build upon the research presented by Wolfrum et al. in their recent publication of a laboratory-scale pretreatment and enzymatic hydrolysis assay, by further developing a more rapid screening process for the determination of composition and reactivity (measures of carbohydrate release and yield) [31]. In their work, a detailed analysis of assay conditions and differences in reactivity results based on differences in feedstock type is presented. Here, we accomplish a more rapid screening method by determining the feasibility of developing multispecies calibrations for composition and reactivity using NIR spectroscopy and partial least squares (PLS) multivariate analysis. Additionally, this work was used to demonstrate the ability to develop these models using a high-throughput form of scanning. Not only does this provide the analyst with a rapid, cost efficient means to predict composition and reactivity for a relatively wide variety of feedstocks simultaneously, but also with the ability to scan large numbers of samples relatively quickly. These methods provide powerful tools for the selection of more promising samples for further research and development.

## Results and discussion

A set of 279 samples was assembled from a large population of feedstock samples to develop broad-based multi-feedstock models for composition and reactivity. This population consisted of the major feedstocks: corn stover (70), sorghum (69), miscanthus (38), switchgrass (20), rice straw (16), and a variety of perennial cool season grasses (58, including wheat straw, wild rye, brome, and fescue). These samples were assembled from a wide variety of independent collections, including samples from well-developed single feedstock calibrations. These single feedstock calibrations were, in some cases, developed over more than a decade and are comprised of samples from a variety of locations across the US, a variety of cultivars or varietals within a specific feedstock, multiple harvest years, and anatomical fractions. When samples were selected from well-developed calibrations, they were chosen from the larger calibration set using principle component analysis (PCA) scoring. This method was employed to ensure the selection of an evenly distributed population representative of the spectral, and therefore compositional, range of the initial population. Samples were also largely selected because they were previously analyzed for chemical composition and in some cases reactivity.

All 279 samples were analyzed for composition; 193 of those samples were analyzed for sugar release (glucose and xylose) and sugar yield from a high-throughput pretreatment and enzymatic hydrolysis assay. One hundred fifty of the 193 samples were previously reported by Wolfrum et al. in their manuscript on reactivity [31]. The populations used for the composition and reactivity models presented in this work are similar but not identical to each other largely because some samples in the biomass composition model did not have enough material for reactivity analysis. Similarly, the sample sets reported here differ slightly from those in the Wolfrum et al. reactivity manuscript simply because we have analyzed additional samples for biomass composition and reactivity as they became available since the Wolfrum et al. manuscript was published.

Based on the preceding explanation of the assembled population, we believed it was suitable to select both calibration and validation sets for composition and reactivity from this population. Specific details for the selection of validation samples from the larger population are further described in the “Methods” section of this paper. However, the validation set does straddle the line between being truly independent for some samples (no relation to the calibration population) and more of a training set for others (some relation to the calibration population). Therefore, it is subsequently referred to as an external validation set.

All 279 samples were scanned using two different NIR spectrometers and three distinct scanning geometries. Samples were scanned on a Thermo Antaris II FT-NIR using the 40-place autosampler (AS) carousel with disposable glass vials and using the spinning ring cup (SRC) attachment with reusable cups possessing optical glass interfaces. Samples were also scanned on a dispersive NIR instrument, Foss XDS Rapid Content analyzer, which also uses sampling cups with optical glass interfaces. These three scanning methods were investigated to compare slower methods of scanning, which use a larger sample size and optical glass containers, with a faster method using containers of lesser quality and sample size. The FT-NIR autosampler data is reported here in detail because it best supports a rapid method for feedstock screening. Results for models built using the other two methods are reported in Additional files 1, 2, 3, and 4 and will be discussed briefly for comparison.

### Composition model

A set of 232 herbaceous feedstock samples, from the assembled population of 279, consisting of the six different herbaceous feedstock species was selected as the calibration set for composition. Feedstocks included in this model were corn stover (56), sorghum (64), switchgrass (16), miscanthus (30), cool season grasses (52), and rice straw (14). A set of 25 external validation samples was also selected and included the six feedstock types: corn stover (4), sorghum (5), switchgrass (2), miscanthus (8), cool season grasses (5), and rice straw (1). Several constituents were available for evaluation; however, glucan, xylan, and lignin, as the three most abundant cell wall components, were the focus of model development [27]. Glucan and xylan content provide information on the major carbohydrates available for bioconversion and lignin content provides information on the level of recalcitrance hindering access to these carbohydrates [28-30]. Ash was also included because of its negative effect on the bioconversion process. However, in contrast to the other modeled constituents which contain organic bonds, ash cannot be directly measured by NIR which measures vibrations in organic bonds. Instead, this inorganic material is indirectly measured by its association or affect on adjacent organic bonds [4].

**Figure 1:**
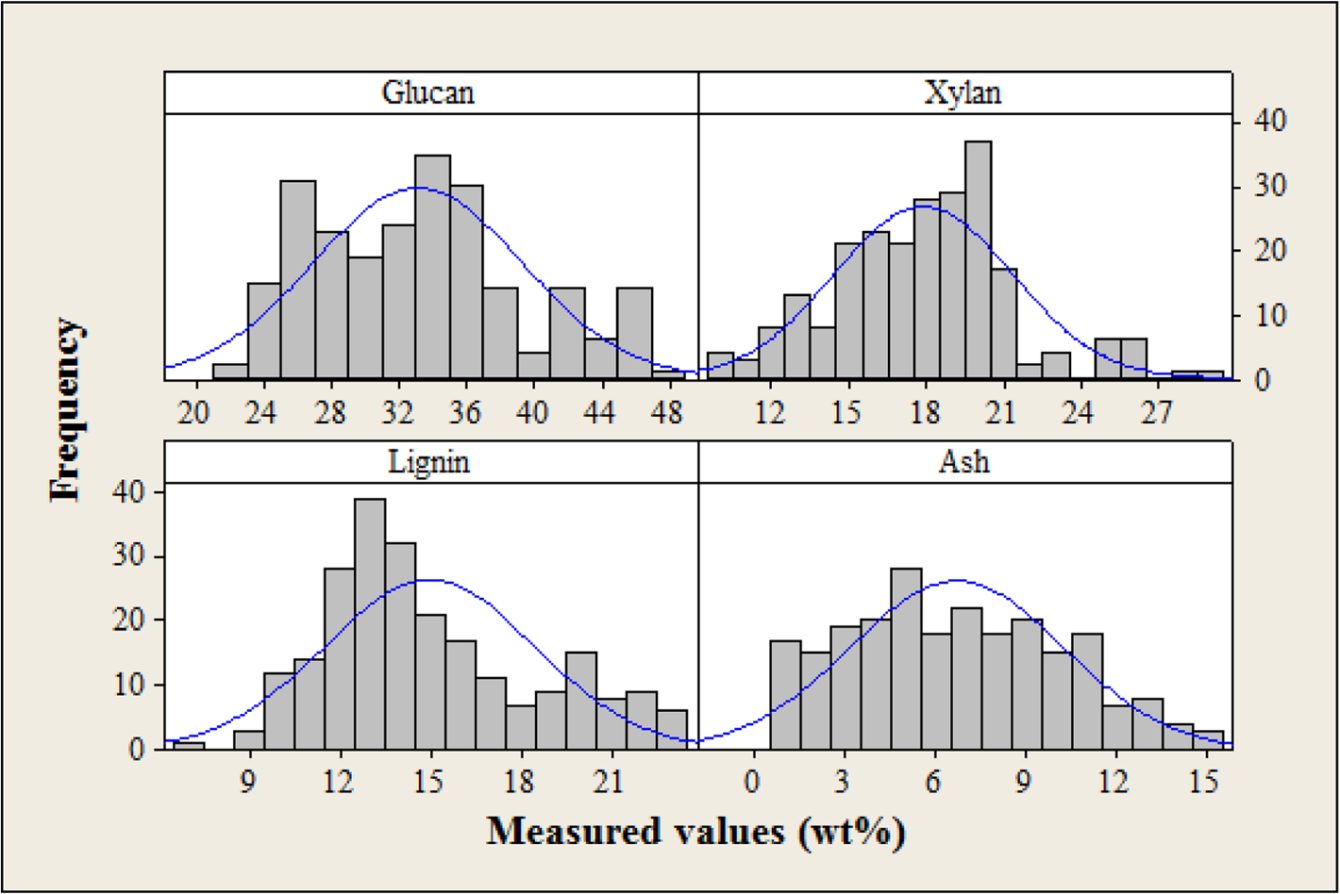
**Histograms for glucan, xylan, lignin, and ash of the 232 sample calibration set.** Composition was measured on a percent dry weight basis. Frequency refers to the number of samples with a given weight percent for each constituent. The blue lines represent normal distributions and are intended to highlight any discrepancy between the histogram and normality. The calibration set does not have normal distribution for any of the constituents, which is not unexpected for a multispecies feedstock population.

Descriptive statistics of these constituents for the calibration and external validation sample sets are reported in Table 1. The broad range of values for each constituent can largely be attributed to the range in feedstock species and cultivar. Histograms for the calibration set are provided in Figure 1 and show the breadth in the range of values. They also show that for some constituents, the majority of the samples fell within a smaller binned range. The blue lines overlaid on each histogram represent normal distributions and are intended to highlight any discrepancy between the histogram and normality. We made no attempts to correct for bimodal (glucan and lignin), skewed (xylan), or uniform distributions.

**Table 1:** **Descriptive statistics of composition for the 232 calibration and the 25 validation sample sets**

	**Calibration**					**Validation**				
	***N***	**Mean**	**SD**	**Min**	**Max**	***N***	**Mean**	**SD**	**Min**	**Max**
Glucan	232	33.2	6.3	21.4	47.8	25	35.0	7.9	23.6	45.6
Xylan	232	17.8	3.4	9.5	28.7	25	17.6	3.0	10.4	21.7
Lignin	232	15.2	3.8	6.7	29.0	25	16.4	4.7	9.5	23.4
Ash	232	6.7	3.6	0.9	16.4	25	6.0	4.1	0.9	16.4

Composition statistics are reported on a dry weight basis. Both calibration and validation sample sets include six herbaceous feedstock types.

*N* number of samples, *SD* standard deviation, *Min* minimum value, *Max* maximum value.

A partial least square two (PLS-2) multivariate calibration model was developed using Thermo FT-NIR autosampler spectral data for the prediction of the four constituents. Spectral data were mathematically preprocessed and the spectral range reduced prior to model development. The spectra were also weighted by one over the standard deviation for each wavenumber (cm^−1^) in the spectral range. The chemical constituents were not weighted. The model was fully cross-validated using the “leave-one-out” method. In this method, a single sample is removed from the model, and the model rebuilt without the sample. The optimal number of factors for the model was determined by comparing the explained variance in the spectral data of the calibration, for all four constituents, to the maxima of the explained variance in the spectral data of the cross-validation. The root-mean-square-error of the calibration (RMSEC) and the root-mean-square-error of the cross-validation (RMSECV) were also used to determine the appropriate number of factors for the model. The number of factors which resulted in RMSEC and RMSECV values that approximated the uncertainties in the primary methods were considered along with the explained variance [32]. RMSEC and RMSECV values were higher than those reported for our primary methods but are consistent with our experience in working with a large variety of feedstock types. In this case, nine factors proved sufficient and possibly conservative, but without danger of over fitting.

**Figure 2:**
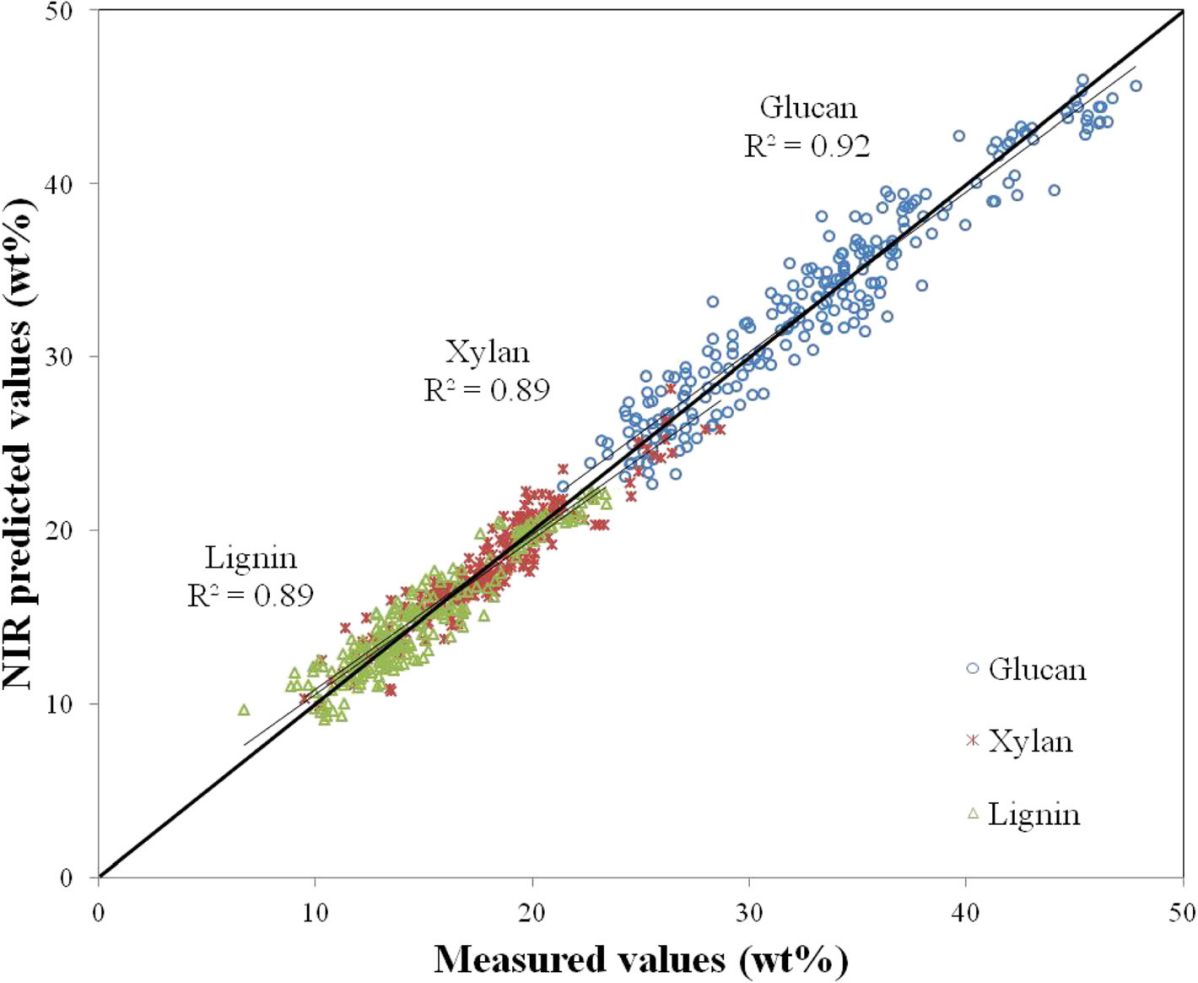
**Predicted versus measured values of glucan, xylan, and lignin for the 232 calibration samples.** The *x*-axis represents constituent values obtained from primary methods measured on a percent dry weight basis (wt%). The *y*-axis represents values for composition obtained by prediction from the PLS-2 calibration equation. Ash is not pictured here for ease of visibility.

Summary statistics for the model are provided in Table 2. This summary includes the RMSEC and the RMSECV. As previously stated, these values approximate the uncertainties in the primary methods of measurement [32]. Also included in the table is the square of the correlation coefficient or coefficient of determination of the cross-validation (*R*^2^). This value is generally lower than for the calibration but gives a better indication of the model’s performance. Slope and intercept are also provided and describe the line of best fit for the cross-validated model. Figure 2 illustrates the good correlations (*R*^2^ > 0.8) between predicted and reference values for glucan, xylan, and lignin in the calibration model. Ash is not depicted for ease of visibility of the other constituents but is reasonably well modeled.

**Table 2:** **Summary statistics for the PLS-2 calibration model for composition using the Thermo FT-NIR spectrometer with autosampler**

**Constituent**	**Samples**	**Factors**	**RMSEC**	**RMSECV**	***R*** ^**2**^	**Slope**	**Intercept**
Glucan	232	9	1.7	1.9	0.91	0.91	2.9
Xylan	232	9	1.1	1.2	0.87	0.87	2.3
Lignin	232	9	1.1	1.2	0.88	0.89	1.7
Ash	232	9	1.3	1.4	0.84	0.84	1.0

RMSECV values are slightly higher than the uncertainty of the primary analytical methods. Slope and intercept describe the line of best fit for cross-validation.

*RMSEC* root-mean-square-error of the calibration model, *RMSECV* root-mean-square-error of cross-validated model, *R* ^*2*^ square of the correlation coefficient of the cross-validated model.

**Figure 3:**
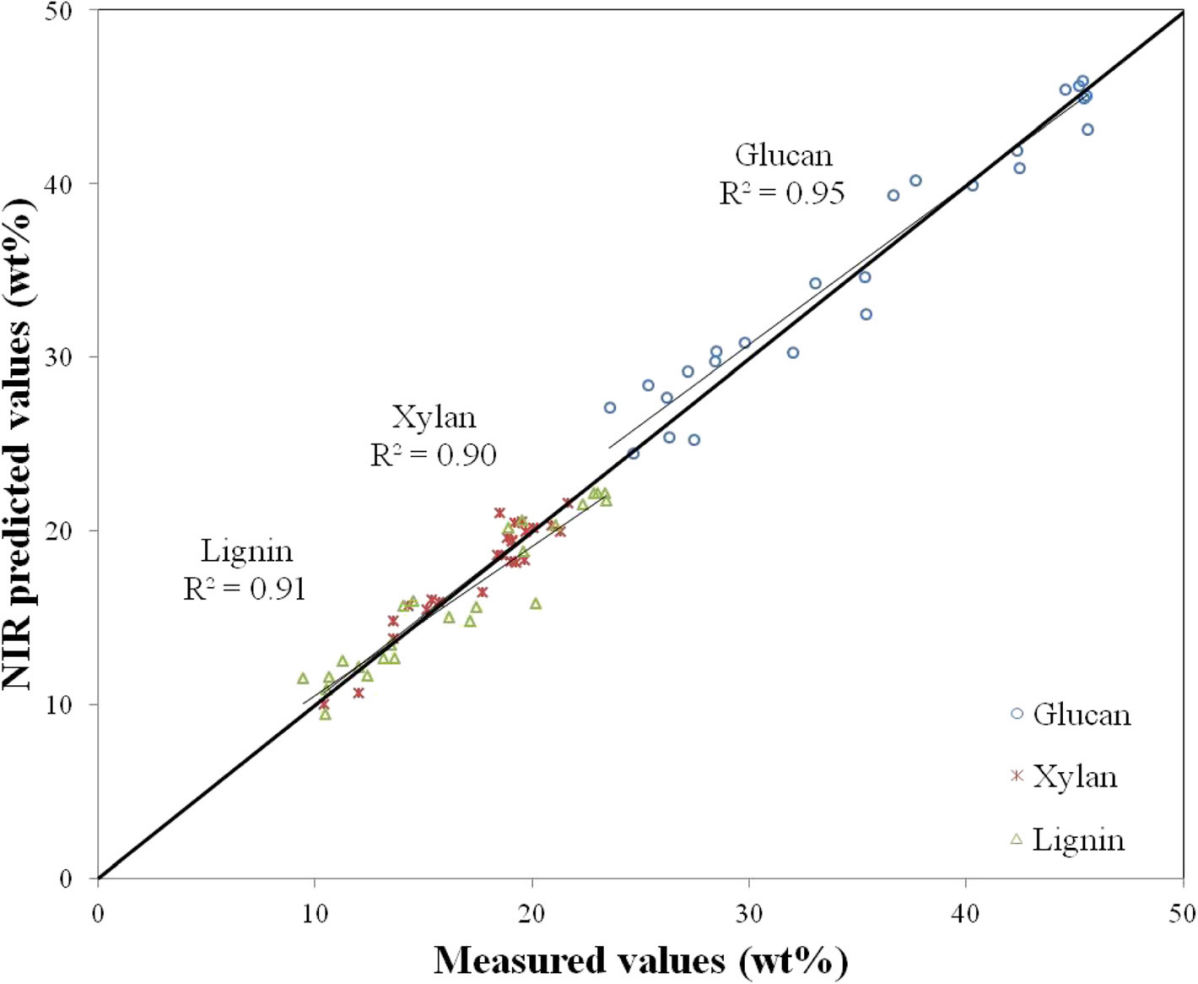
**Predicted versus measured values of glucan, xylan, and lignin for the 25 external validation samples.** The *x*-axis represents constituent values obtained from primary methods measured on a percent dry weight basis (wt%). The *y*-axis represents values for composition obtained by prediction form the PLS-2 calibration equation. Ash is not pictured here for ease of visibility. The external validation samples were not used to build the calibration model.

The 25 external validation samples were also predicted using the calibration model. Summary statistics for the prediction of these samples are provided in Table 3. This summary includes the root-mean-square-error of the prediction (RMSEP), which approximates the uncertainties in the primary methods of measurement. Also included in the table are the square of the correlation coefficients of the external validation (*R*^2^). Slope and intercept are also provided and describe the line of best fit for the external validation. Figure 3 further illustrates the good correlations (*R*^2^ > 0.8) between predicted and reference values for glucan, xylan, and lignin from the calibration model. Again, ash is not depicted for ease of visibility of the other constituents. The validation set is well predicted by the model which further demonstrates the utility of a multi-feedstock broad-based model for composition. This model performs better or similarly to the corn stover model reported by Wolfrum and Sluiter [6] when comparing values of *R*^2^ and RMSECV for the cross-validated model for glucan, xylan, and lignin [6]. The model does have slightly lower values for *R*^2^ and higher values for RMSECV when compared to the sorghum cross-validated model reported by Wolfrum et al. [33]. A review of the literature from 2010 to the present suggests this model is similar to single feedstock models for glucan, xylan, and lignin when comparing values of *R*^2^ and RMSECV for the cross-validated model [13,15,20,34-38]. This model’s performance is also very similar to the multispecies feedstock models reported by da Silva Perez et al. 2010 and Monono et al. [18,19].

**Table 3:** **Summary statistics for external validation of the PLS-2 calibration model for composition**

**Constituent**	**Samples**	**Factors**	**RMSEP**	***R*** ^**2**^	**Slope**	**Intercept**
Glucan	25	9	1.8	0.95	0.92	3.1
Xylan	25	9	1.0	0.90	0.97	0.6
Lignin	25	9	1.5	0.91	0.86	2.0
Ash	25	9	1.3	0.89	0.90	0.3

The RMSEP values are slightly higher than the uncertainty of the primary analytical methods. The slope and intercept describe the line of best fit for these samples.

*RMSEP* root-mean-square-error of prediction, *R* ^*2*^ square of the correlation coefficient for the external validation predictions.

### Release and yield models

**Figure 4:**
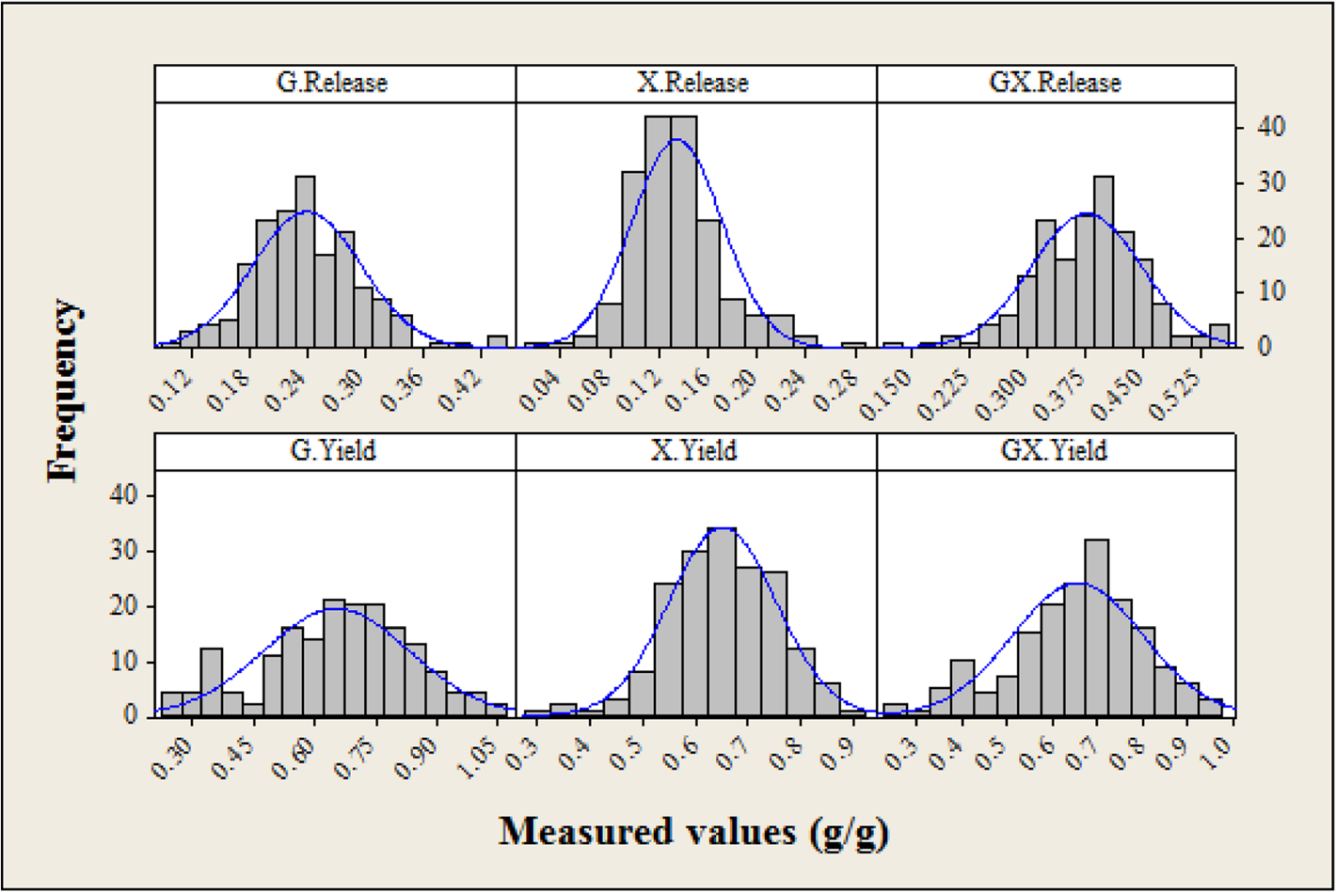
**Histograms of glucose and xylose release and yield for the six calibration sets.** G.Release and X.Release refer to the individual carbohydrates released following pretreatment and enzymatic hydrolysis. GX.Release refers to the sum of glucose and xylose released following pretreatment and enzymatic hydrolysis. Release was measured as the mass of carbohydrate release per unit of dry biomass in grams per gram. G.Yield refers to the ratio of glucose release (as previously defined and anhydro corrected) to the glucan and sucrose mass fraction in the feedstock. X.Yield refers to the ratio of xylose release to the xylan mass fraction in the feedstock. GX.Yield refers to the sum of the two carbohydrate yields as previously explained. Frequency on the *y*-axis refers to the number of samples with a given value for each constituent. The blue lines represent normal distributions and are intended to highlight any discrepancy between the histogram and normality. The calibration set does not have a normal distribution for any of the constituents, which is not unexpected for a multispecies feedstock population.

A set of 164 to 167 feedstock samples, depending on the constituent modeled, consisting of six to seven different herbaceous and two woody feedstocks were selected as the calibration set for the carbohydrate release and yield models. Feedstocks included in these models were corn stover, sorghum, switchgrass, miscanthus, a variety of cool season grasses, sugarcane bagasse, rice straw, pine, and poplar. A single set of 18 validation samples was selected for external validation of the models and included sorghum, corn stover, miscanthus, cool season grasses, switchgrass, and poplar feedstock types. The following constituents were modeled as a combined result of pretreatment and enzymatic hydrolysis: glucose released (G.Release), xylose released (X.Release), the sum of glucose and xylose released (GX.Release), glucan yield (G.Yield), xylan yield (X.Yield), and the sum of glucan and xylan yields (GX.Yield). Descriptive statistics of these constituents for the calibration and external validation sample sets are reported in Table 4. Histograms are also provided to give an alternative view of the range in values of the six constituents for the calibration set (Figure 4).

**Table 4:** **Descriptive statistics for carbohydrate release and yield following pretreatment and enzymatic hydrolysis for calibration and validation sample sets**

	**Calibration**					**Validation**				
	***N***	**Mean**	**SD**	**Min**	**Max**	***N***	**Mean**	**SD**	**Min**	**Max**
GX.Release	166	0.38	0.07	0.13	0.56	18	0.39	0.12	0.20	0.66
G.Release	167	0.24	0.06	0.10	0.44	18	0.25	0.09	0.12	0.48
X.Release	167	0.13	0.04	0.03	0.24	18	0.14	0.05	0.07	0.28
GX.Yield	164	0.65	0.14	0.27	0.9	18	0.65	0.21	0.28	0.97
G.Yield	165	0.65	0.18	0.23	1.02	18	0.64	0.25	0.24	1.00
X.Yield	165	0.65	0.10	0.32	0.91	18	0.66	0.13	0.37	0.96

G.Release and X.Release are glucose and xylose release (grams per gram feedstock). GX.Release is the release of both carbohydrates. G.Yield and X.Yield are the yields of glucan or xylan, while GX.Yield refers to the sum of the two carbohydrate yields. Yield data are expressed as the fraction of structural carbohydrate released into solution.

Partial least square one and two (PLS-1 and PLS-2) multivariate calibration models were developed using Thermo FT-NIR autosampler spectral data for the prediction of the six variables. PLS-1 was used to model GX.Release and GX.Yield, while PLS-2 was used to model G.Release and X.Release in one model, and G.Yield and X.Yield in another. Spectral data were mathematically preprocessed and the spectral range reduced prior to model development. The spectra were also weighted or standardized one over the standard deviation for each wavenumber (cm^−1^) in the spectral range. The chemical constituents were weighted in the PLS-2 models, one over the standard deviation (1/SD). All models were fully cross-validated using the “leave-one-out” method as previously described. The optimal number of factors for each model was also determined as previously described by comparing the explained variance in the spectral data of the calibration to the explained variance in the spectral data of the cross-validation, for each constituent. The appropriate numbers of factors determined for each model are listed in Table 5.

**Table 5:** **Summary statistics for calibration models for carbohydrate release and yield following pretreatment and enzymatic hydrolysis**

**Constituent**	**Samples**	**Factors**	**RMSEC**	**RMSECV**	***R*** ^**2**^	**Slope**	**Intercept**
GX.Release	166	9	0.03	0.03	0.78	0.80	0.07
G.Release	167	11	0.02	0.03	0.80	0.81	0.05
X.Release	167	11	0.01	0.01	0.82	0.84	0.02
GX.Yield	164	8	0.05	0.06	0.84	0.85	0.10
G.Yield	165	8	0.06	0.07	0.84	0.85	0.10
X.Yield	165	8	0.05	0.05	0.70	0.73	0.18

GX.Release and GX.Yield are separate PLS-1 models. G.Release and X.Release, and G.Yield and X.Yield are combined PLS-2 calibration models. “Factors:” optimal number of factors for the model. RMSECV values are slightly higher than the uncertainties of the primary analytical methods. The slope and intercept describe the line of best fit for cross-validation.

*RMSEC* root-mean-square-error of the calibration model, *RMSECV* root-mean-square-error of cross-validated model, *R* ^*2*^ square of the correlation coefficient of the cross-validated model.

**Figure 5:**
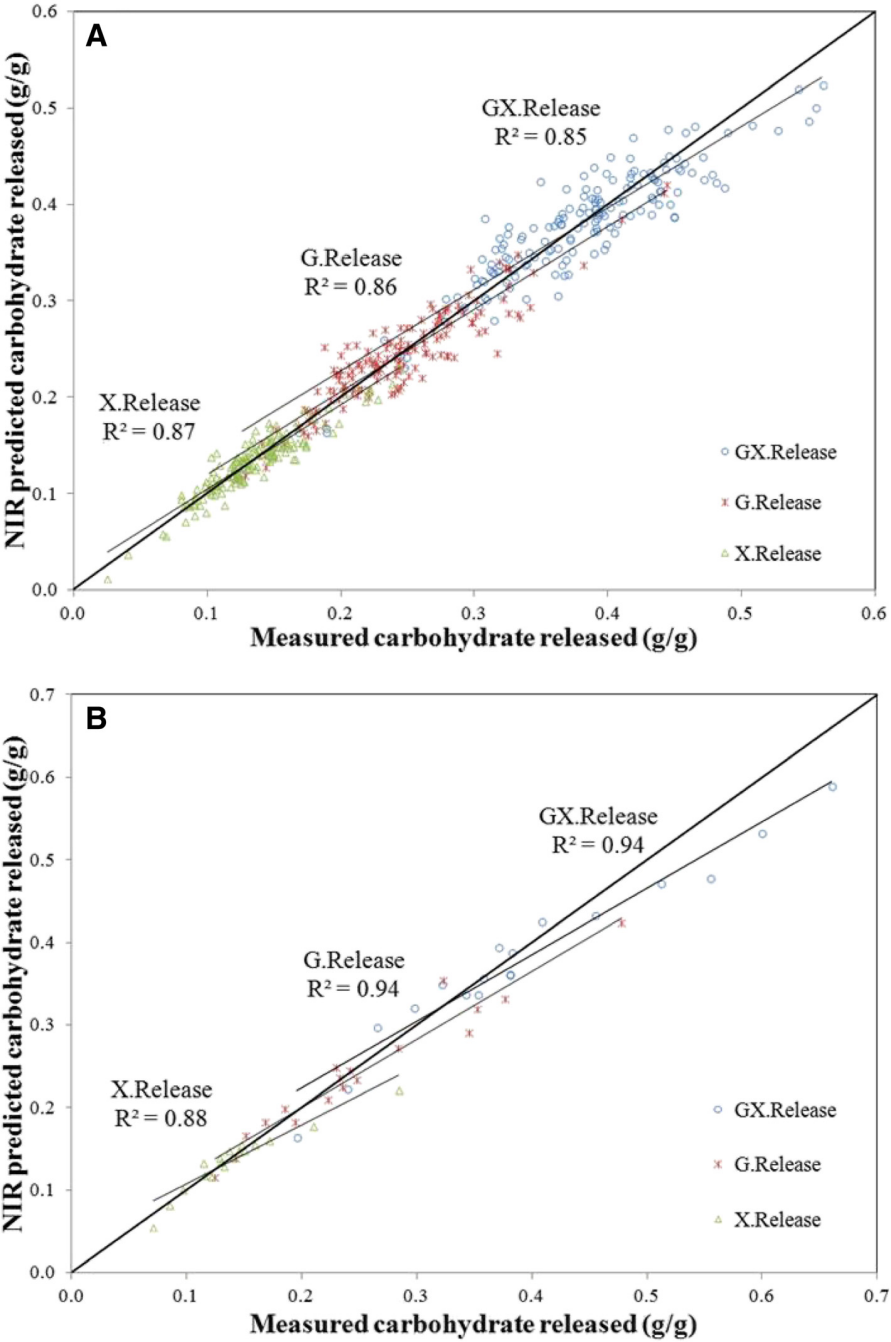
**Predicted versus measured values of carbohydrate release following pretreatment and enzymatic hydrolysis.** The *x*-axis represents release values obtained from primary methods measured in grams per gram. The *y*-axis represents values for carbohydrates released in grams per gram as predicted on the PLS-2 calibration equation for glucose and xylose separately (G.Release and X.Release), or as predicted on the PLS-1 calibration equation for the sum of glucose and xylose released (GX.Release). Predictions are from the calibration models. **(A)** Predicted versus measured values of carbohydrate release for the calibration samples. **(B)** Predicted versus measured values of carbohydrate release for the 18 external validation samples.

**Figure 6:**
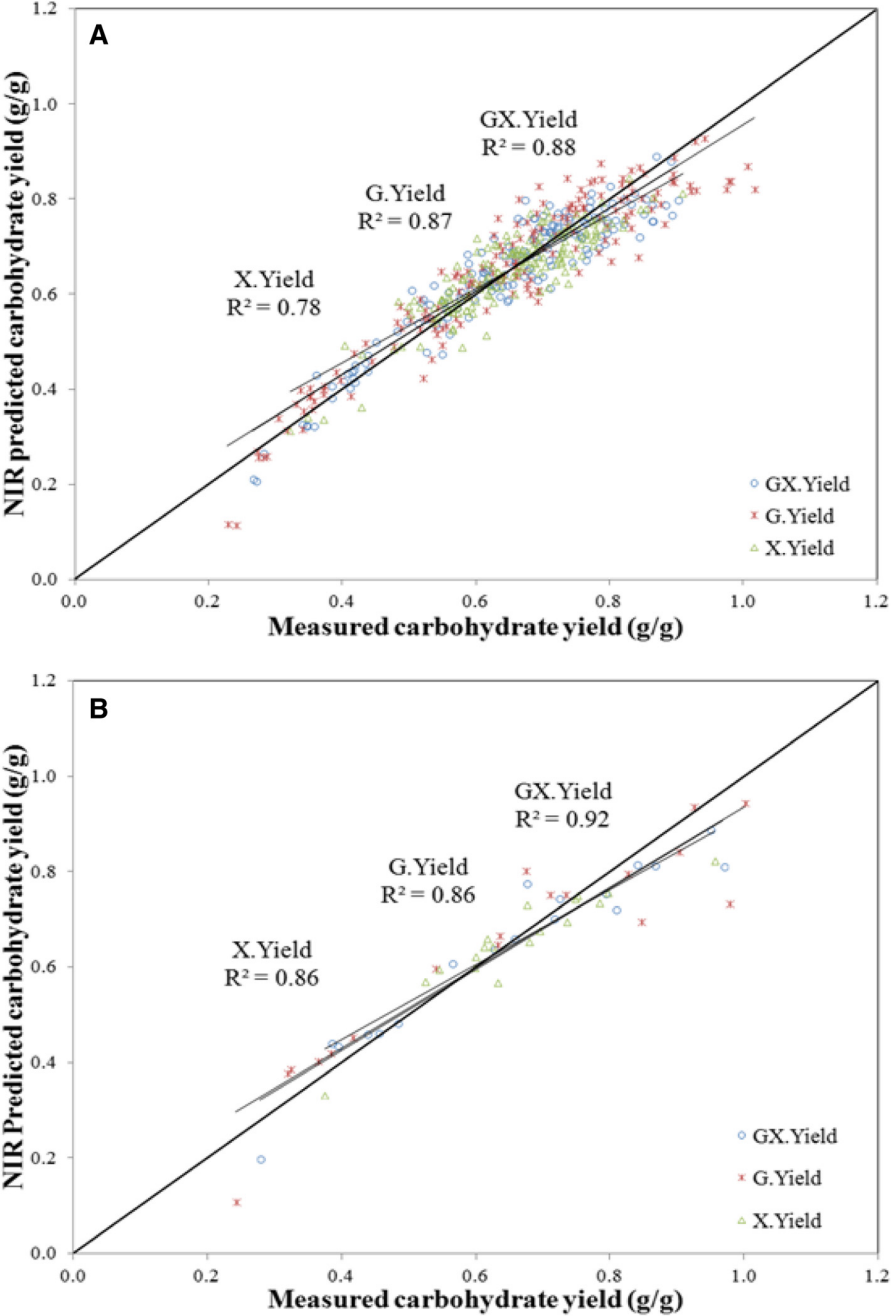
**Predicted versus measured values of carbohydrate yield following pretreatment and enzymatic hydrolysis.** The *x*-axis represents yield values obtained from primary methods measured in grams per gram. The *y*-axis represents values for carbohydrate yield in grams per gram as predicted on the PLS-2 calibration equation for glucan and xylan separately (G.Yield and X.Yield), or as predicted on the PLS-1 calibration equation for the sum of glucan and xylan yield (GX.Yield). Predictions are from the calibration models. **(A)** Predicted versus measured values of carbohydrate yield for the calibration samples. **(B)** Predicted versus measured values of carbohydrate yield for the 18 external validation samples.

Table 5 also includes summary statistics for the four individual release and yield models. This summary includes values for RMSEC and RMSECV. The values reported here are comparable to the uncertainties reported for the bench top total assay [31]. Also included in the table are *R*^2^ values for the cross-validation. Again, this value is generally lower than for the calibration but gives a better indication of the model’s performance. Slope and intercept are also provided and describe the line of best fit for the cross-validated model. Figures 5A and 6B illustrate the correlations between predicted and reference values for the release and yield calibration models, respectively. Both the release and yield models have good correlations (*R*^2^ > 0.8, except X.Yield which is 0.78) and uncertainties that approximate errors in the total assay. Uncertainties for the yield models are twice those of the release models but also include the uncertainties from the wet chemistry.

The 18 external validation samples were predicted on the previously described calibration models, and summary statistics for the prediction of these samples are provided in Table 6. This summary includes RMSEP and *R*^2^ for the external validation set. Slope and intercept are also provided for validation. Figures 5B and 6B further illustrate the correlations between predicted and reference values for release and yield of the validation set, respectively. Both sets of models, release and yield, predict the 18 external validation samples reasonably well, though both models tend to under estimate accessible carbohydrates at high reactivity. Nonetheless, these models are quite valuable in their ability to separate samples into low, medium, and high release and low, medium, and high yield.

**Table 6:** **Summary statistics for external validation of the PLS-1 and PLS-2 calibration models for carbohydrate release and yield**

**Constituent**	**Samples**	**Factors**	**RMSEP**	***R*** ^**2**^	**Slope**	**Intercept**
GX.Release	18	9	0.04	0.94	0.81	0.06
G.Release	18	11	0.03	0.94	0.82	0.04
X.Release	18	11	0.02	0.88	0.71	0.04
GX.Yield	18	8	0.06	0.92	0.85	0.09
G.Yield	18	8	0.09	0.86	0.84	0.09
X.Yield	18	8	0.05	0.86	0.79	0.13

External validation samples were predicted using the following calibration models: the PLS-1 GX.Release and GX.Yield calibration models, the PLS-2 G.Release and X.Release calibration model, and the PLS-2 G.Yield and X.Yield calibration model. “Factors:” the optimal number of factors for the model. The RMSEP values are higher than the uncertainty of the primary analytical methods.

*RMSEP* root-mean-square-error of the prediction of the validation samples, *R* ^*2*^ square of the correlation coefficient of the predicted samples.

### Scanning method comparison

Calibration models for composition and reactivity, as previously described using Thermo FT-NIR autosampler spectra, were developed using Foss XDS spectra and Thermo FT-NIR SRC spectra. Calibration models using SRC and XDS spectra were not individually optimized based on the specific scanning geometry used. Instead, SRC and XDS calibration models were developed using the exact same calibration and validation sample sets, spectral pretreatments, and PLS modeling parameters as those previously described for the Thermo autosampler. The full spectral range (400 to 2,500 nm) was used for Foss XDS model development. A reduced wavelength range (1,000 to 2,500 nm) did not provide superior results, as demonstrated by RMSEC, RMSECV, RMSEP, and *R*^2^ values associated with each of these statistics (data not shown). Summary statistics for models developed using Foss XDS spectra can be found in Additional files 1 and 3. Summary statistics for models developed using Thermo FT-NIR SRC spectra can be found in Additional files 2 and 4.

The purpose of this particular experiment was to determine if the higher throughput scanning method of the Thermo FT-NIR autosampler, which uses disposable glass vials instead of cups with optical glass interfaces, was inferior to traditional methods of scanning. These slower manual methods, Foss XDS ring cups and the Thermo FT-NIR SRC, might provide either more spectral information due to a larger scanning window or better spectral information through the reduction of spectral noise or scatter by use of higher quality optical glass. Based on this particular comparison, there are no statistically significant differences (*p* = 0.05) between PLS-2 models for composition when comparing calibration, cross-validation, and external validation statistics (*R*^2^ and RMSE). This is also true for the PLS-1 and PLS-2 models for reactivity. Furthermore, a *t*-test (*p* = 0.05) comparing the external validation predictions, from each of the scanning methods, to the reference values of the external validation set, showed no statistically significant differences for either composition or reactivity (data not shown). This suggests that the autosampler is not an inferior method of scanning despite its use of a low quality scanning interface. It is however important to note that modeling with the ASRS data did require the use of one or two additional factors.

Using the Thermo FT-NIR autosampler for NIR rapid analysis allows for a total analysis time of around 5 to 10 min per sample. The autosampler offers the advantage of simultaneous sample preparation and scanning. When using the Thermo FT-NIR SRC or Foss XDS, this number increases as sample number increases because simultaneous sample preparation and scanning is limited. Estimating total analysis time using NIR rapid analysis can be misleading. While it takes less than a minute for either instrument or scanning method to perform a scan, there are other necessary steps involved in the process. This includes sample preparation, scanning and prediction as well as scanning and prediction of reference samples to ensure instrument stability.

With respect to bench top methods, the total analysis time per sample is difficult and potentially misleading to estimate because analyses are preformed on multiple samples at one time. The size of a sample set can vary, and different analyses are performed by different analysts essentially concurrently. Using the National Renewable Energy Laboratory (NREL) published methods for compositional analysis and the reactivity assay, for pretreatment and enzymatic hydrolysis as outlined in Wolfrum et al. the total time can be estimated at 10 to 12 days per sample set [31,39]. Composition and reactivity equate to 7 to 10 days per sample set based on analyst time. However, the enzymatic hydrolysis requires 7 days for complete hydrolysis of the substrate, which requires little analyst time and is independent of batch size. Regardless of the ability to more accurately estimate the time required for bench top analyses, the ability to develop multivariate calibrations using NIR spectroscopy imparts significant savings on analysis time and therefore, cost once the models have been developed.

## Conclusion

We have demonstrated that it is possible to build effective broad-based multispecies-feedstock models for composition and reactivity using near-infrared (NIR) spectroscopy and partial least squares (PLS) multivariate analysis. These models represent no less than six feedstock types comprised of multiple cultivars, harvest years, locations, and anatomical fractions. The model for composition is useful for predicting glucan, xylan, lignin, and ash with good uncertainties. The release and yield models have higher uncertainties than the model for composition. However, these reactivity models are useful for rapidly screening sample populations to separate samples into low, medium, and high reactivity based on carbohydrate release and yield. Therefore, unusual samples can be identified for further investigation.

The results from this work also demonstrate that it is possible to build effective models using spectral data obtain from a higher throughput method of scanning. Though the use of this method required a low quality borosilicate vial for scanning, our results have shown that it does not significantly affect the quality or predictive ability of the resulting model. These multispecies-feedstock models for composition and reactivity combined with a higher throughput form of scanning provide researchers with a powerful set of tools to rapidly identify more promising samples for further development as biofuels feedstocks.

## Methods

### Sample selection

The 279 samples with chemical composition were divided into samples for calibration and validation. Nine samples were removed from the population prior to calibration selection. These samples consisted of feedstocks which were not well represented in number which included samples of poplar, pine, and sugarcane bagasse. Once these samples were eliminated, 245 calibration samples were selected from the resulting population of 270 samples using the Kennard-Stone algorithm applied to preprocessed spectral data across two principal components (PC) [40]. This algorithm selects a pre-determined number of samples from a population based on spectral variation across a select number of PC. This left 25 samples that were used as the external validation set. The 25 samples were well distributed across the six herbaceous feedstock species.

The overlapping 193 samples with chemical composition and reactivity data were also divided into samples for calibration and external validation. The same algorithm was applied to the preprocessed spectral data, which allowed for the selection of 175 samples across two PCs. This left 18 samples that were used as an external validation set and were well represented in number across feedstock type.

The number of samples in each of the previously described calibration sets for composition and reactivity were further reduced by the removal of sample outliers. Base models were developed for the full calibration set, and from these models, outliers were determined. To identify outliers, we first calculated the difference between the actual or reference value and the predicted values, and then normalized these differences by dividing them by the RMSEC of that constituent for the initial calibration model. For example, given a single sample and the constituent glucan, the following equation was used:

$$ \mathrm{Glucan}\;\mathrm{Error}=\frac{\mathrm{abs}\left({Y}_{\mathrm{Ref}}^{\mathrm{G}}-{Y}_{\Pr \mathrm{e}}^{\mathrm{G}}\right)}{{\mathrm{RMSEC}}^{\mathrm{G}}} $$

where “abs” refers to the absolute value of the difference, ${Y}_{\mathrm{Ref}}^{\mathrm{G}}$ is the reference glucan value, ${Y}_{\Pr \mathrm{e}}^{\mathrm{G}}$ is the glucan value predicted by the model, and the RMSEC^G^ is the root-mean-square-error of calibration for glucan of the model. We then compared these normalized values to a “cut off” value, in this case 1.5 for composition and 2.0 for the release and yield models.

For a single sample, this calculation was preformed for each constituent modeled (e.g., xylan, lignin, GX.Yield, etc.) and the result of that calculation compared to the cutoff values 1.5 or 2.0. In most cases, samples with calculated values greater than the cutoff values were omitted as outliers. For the composition model, the results of the error calculation for glucan, xylan, and lignin were averaged for a given sample and then compared to 1.5. Ash outliers were removed separately using the same method.

### Composition and reactivity analysis

All samples were previously analyzed for chemical composition using the publicly available NREL suite of laboratory analytical procedures: www.nrel.gov/biomass/analytical_procedures.html [39]. The history and typical uncertainties related to these methods have been published elsewhere [32,41]. These methods included a two phase solvent extraction by water and then ethanol, followed by a two-stage sulfuric acid hydrolysis. Ash and moisture were determined gravimetrically and all measured constituents were corrected to a dry weight basis. Prior to compositional analysis samples were dried to less than 10% moisture and milled to a 2-mm particle size using a bench top or Wiley mill. Constituents measured were total ash (structural and non-structural), protein (structural and non-structural), sucrose, water extractives, ethanol extractives, starch, lignin, glucan, xylan, galactan, arabinan, fructan or mannan, and acetic acid.

A subset of 193 samples were analyzed for glucose and xylose release and yield in a rapid reactivity assay developed by Wolfrum et al. 150 of those samples being previously reported in that manuscript [31]. This assay included a dilute acid pretreatment (PT) for the release of carbohydrates by automated solvent extractor (ASE 350, Dionex, Sunnyvale, CA) followed by enzymatic hydrolysis (EH) of the remaining solid sample for the release of additional carbohydrates. The specific methods used from Wolfrum et al. were those developed for “optimal pretreatment conditions for screening” which held the pretreatment conditions constant [31]. The dilute acid pretreatment used a constant temperature of 130°C for 7 min, 3.0 g sample, and 30 mL of a 1% sulfuric acid solution. The enzymatic hydrolysis method was similar to the NREL LAP, “Enzymatic Hydrolysis of Lignocellulosic Biomass” [39]. Both release and yield measurements reflect the sum of each carbohydrate obtained after the combined or total assay (PT plus EH). Glucose and xylose release, as a result of pretreatment and subsequent enzymatic hydrolysis, was defined as the mass of carbohydrate released per unit of dry biomass. The xylan yield was defined as the ratio of xylose release to the xylan mass fraction in the feedstock, with anhydro correction for conversion of xylose to xylan. The glucan yield was defined as the ratio of glucose release to the glucose and sucrose mass fraction in the feedstock, with anhydro correction for conversion of glucose to glucan. A more detailed description of these calculations and the assumptions inherent in them is provided by Wolfrum et al. [31].

### NIRS analysis

All samples scanned were milled to a 2-mm particle size and dried to less than 10% moisture. Each sample was scanned in duplicate from two separate samplings and the duplicate spectra averaged. Samples were scanned on both FT and dispersive NIR instruments: Thermo Antaris II FT-NIR and Foss XDS Rapid Content Analyzer. Samples scanned on the FT-NIR were scanned using two different scanning attachments, the Autosampler RS and the spinning ring cups. The autosampler uses commercially available, disposable, borosilicate 2 dram glass vials, while the spinning ring cups are Thermo specific, reusable, and constructed from optical glass. Both scanning geometries averaged 128 scans per sample using the wave number range of 12,000 to 3,300 with a resolution of 8 cm^−1^ (3.857 cm^−1^ data spacing). Samples scanned on the Foss XDS used either the ring or quarter sampling cups both constructed with optical glass. Samples scanned on the Foss averaged 32 scans per sample using the wavelength range of 400 to 2,500 with 0.5 nm data spacing.

### Statistical analysis

Sample spectra were mathematically preprocessed and the spectral range reduced prior to model development. Spectra were first transformed using the standard-normal-variate (SNV) for scatter correction. Then, a Savitzky-Golay first derivative, second order polynomial, with 21 point smoothing, was applied to correct baseline variation. The spectral range was then reduced to 4,000 to 8,998 cm^−1^ to remove spectral regions corresponding to increased variations in the signal response but with no significance for improved modeling of composition and reactivity.

Partial least squares (PLS) multivariate calibrations were developed using both Unscrambler X 10.3 (Camo USA) and R open source software (http://www.r-project.org) [42]. Using these software packages two different types of PLS models were developed: PLS-1 and PLS-2. PLS-1 models relate a single dependent variable such as lignin to a function of the dependent variable, the NIR spectra. PLS-2 models relate more than one dependent variable such as lignin, glucan, and xylan to a function of the dependent variable, the spectra. Therefore, in this case, PLS-1 models predict a single constituent while PLS-2 models predict multiple constituents. PLS-1 models were developed for the sum of glucose and xylose released from pretreatment and enzymatic hydrolysis, and the sum of glucan and xylan yielded from pretreatment and enzymatic hydrolysis. PLS-2 models were developed for composition (glucan, xylan, lignin, and ash) and release of glucose and xylose as measured independently as well as yield of glucan and xylan as measured independently.

## Additional files

Additional file 1:
**Summary statistics for the PLS-2 calibration model for glucan, xylan, lignin, and ash content and external validation statistics using spectra from the Foss XDS.** RMSEC describes the root-mean-square-error of the calibration model, while the RMSECV describes the cross-validated model. RMSECV values are higher than the primary methods of uncertainty, but closely resemble them. *R* ^2^ is the square of the correlation coefficient of the cross-validated model. This value is generally lower than for the calibration model but gives a better indication of the models performance. The slope and intercept describe the line of best fit for the cross-validated model. Twenty-five external validation samples were predicted using the nine factor calibration model for glucan, xylan, lignin, and ash. RMSEP describes the root-mean-square-error of prediction. These values are higher than the primary methods of uncertainty, but closely resemble them. *R* ^2^ is the square of the correlation coefficient of the externally validated set. The slope and intercept describe the line of best fit for the externally validated set. Additional file 2:
**Summary statistics for the PLS-2 calibration model for glucan, xylan, lignin, and ash content and external validation statistics using spectra from the Thermo FT-NIR SRC.** RMSEC describes the root-mean-square-error of the calibration model, while the RMSECV describes the cross-validated model. RMSECV values are higher than the primary methods of uncertainty, but closely resemble them. *R* ^2^ is the square of the correlation coefficient of the cross-validated model. This value is generally lower than for the calibration model but gives a better indication of the models performance. The slope and intercept describe the line of best fit for the cross-validated model. Twenty-five external validation samples were predicted using the nine factor calibration model for glucan, xylan, lignin, and ash. RMSEP describes the root-mean-square-error of prediction. These values are higher than the primary methods of uncertainty, but closely resemble them. *R* ^2^ is the square of the correlation coefficient of the externally validated set. The slope and intercept describe the line of best fit for the externally validated set. Additional file 3:
**Summary statistics for calibration models for carbohydrate release and yield following pretreatment and enzymatic hydrolysis including external validation statistics using spectra from the Foss XDS.** GX.Release and GX.Yield describe PLS-1 model statistics while G.Release and X.Release were combined for PLS-2 calibration. G.Yield and X.Yield were also combined for PLS-2 calibration. The “Factors” column lists the number of factors used to build the model. RMSEC describes the root-mean-square-error of the calibration model, while the RMSECV describes the cross-validated model. RMSECV values are higher than the primary methods of uncertainty. This is particularly true for the yield models because they reflect the accumulated uncertainty from composition, pretreatment, and enzymatic hydrolysis measurements. *R* ^2^ is the square of the correlation coefficient of the cross-validated model. This value is generally lower than for the calibration model but gives a better indication of the model’s performance. The slope and intercept describe the line of best fit for the cross-validated model. In the external validation table, GX.Release and GX.Yield describe PLS-1 model statistics while G.Release and X.Release were combined for PLS-2 calibration. The “Factors” column lists the number of factors used for prediction. RMSEP describes the root-mean-square-error of the prediction: the 18 validation samples predicted on the calibration model. These values are higher than the primary methods of uncertainty. This is particularly true for the yield models because they reflect the accumulated uncertainty from composition, pretreatment, and enzymatic hydrolysis measurements. *R* ^2^ is the square of the correlation coefficient of the externally validated set. The slope and intercept describe the line of best fit for the external validation. Additional file 4:
**Summary statistics for calibration models for carbohydrate release and yield following pretreatment and enzymatic hydrolysis including external validation statistics using spectra from the Thermo FT-NIR SRC.** GX.Release and GX.Yield describe PLS-1 model statistics, while G.Release and X.Release were combined for PLS-2 calibration. G.Yield and X.Yield were also combined for PLS-2 calibration. The “Factors” column lists the number of factors used to build the model. RMSEC describes the root-mean-square-error of the calibration model, while the RMSECV describes the cross-validated model. RMSECV values are higher than the primary methods of uncertainty. This is particularly true for the yield models because they reflect the accumulated uncertainty from composition, pretreatment, and enzymatic hydrolysis measurements. *R* ^2^ is the square of the correlation coefficient of the cross-validated model. This value is generally lower than for the calibration model but gives a better indication of the model’s performance. The slope and intercept describe the line of best fit for the cross-validated model. In the external validation table, GX.Release and GX.Yield describe PLS-1 model statistics while G.Release and X.Release were combined for PLS-2 calibration. The “Factors” column lists the number of factors used for prediction. RMSEP describes the root-mean-square-error of the prediction: the 18 validation samples predicted on the calibration model. These values are higher than the primary methods of uncertainty. This is particularly true for the yield models because they reflect the accumulated uncertainty from composition, pretreatment, and enzymatic hydrolysis. The slope and intercept describe the line of best fit for the externally validated set.

## Abbreviations

AS: Autosampler
ASE: Automated solvent extractor
FT-NIR: Fourier transform near-infrared
NIR: Near-infrared
NREL: National Renewable Energy Laboratory
PC: Principal component
PCA: Principal component analysis
PLS: Partial least squares
*R*^2^: Square of the correlation coefficient
RMSEC: Root-mean-square-error of the calibration
RMSECV: Root-mean-square-error of the cross-validation
RMSEP: Root-mean-square-error of the prediction
SD: Standard deviation
SNV: Standard-normal-variate
SRC: Spinning ring cup

## References

[1] Lupoi JS, Singh S, Simmons BA, Henry RJ (2013). Assessment of lignocellulosic biomass using analytical spectroscopy: an evolution to high-throughput techniques. BioEnergy Res.

[2] Sims REH, Mabee W, Saddler JN, Taylor M (2010). An overview of second generation biofuel technologies. Bioresour Technol.

[3] Jimaré Benito MT, Bosch Ojeda C, Sanchez Rojas F (2008). Process analytical chemistry: applications of near infrared spectrometry in environmental and food analysis: an overview. Appl Spectrosc Rev.

[4] Hames B, Thomas S, Sluiter A. Rapid Biomass Analysis. In Biotechnology for Fuels and Chemicals: the Twenty-Fourth Symposium. Volume 105. Edited by Brian H. Davison, James W. Lee, Mark Finkelstein JDM. Humana Press; 2003:5–16.

[5] Sluiter A, Wolfrum E (2013). Near infrared calibration models for pre-treated corn stover slurry solids, isolated and in situ. J Near Infrared Spectrosc.

[6] Wolfrum EJ, Sluiter AD (2009). Improved multivariate calibration models for corn stover feedstock and dilute-acid pretreated corn stover. Cellulose.

[7] Decker SR, Brunecky R, Tucker MP, Himmel ME, Selig MJ (2009). High-throughput screening techniques for biomass conversion. BioEnergy Res.

[8] DeMartini JD, Studer MH, Wyman CE (2011). Small-scale and automatable high-throughput compositional analysis of biomass. Biotechnol Bioeng.

[9] Chundawat SPS, Balan V, Dale BE (2008). High-throughput microplate technique for enzymatic hydrolysis of lignocellulosic biomass. Biotechnol Bioeng.

[10] Williams P (2013). Tutorial: calibration development and evaluation methods B. Set-up and evaluation. NIR News.

[11] Williams P (2013). Tutorial: calibration development and evaluation methods A. Basics. NIR News.

[12] Huang J, Xia T, Li A, Yu B, Li Q, Tu Y (2012). A rapid and consistent near infrared spectroscopic assay for biomass enzymatic digestibility upon various physical and chemical pretreatments in Miscanthus. Bioresour Technol.

[13] Vogel KP, Dien BS, Jung HG, Casler MD, Masterson SD, Mitchell RB (2010). Quantifying actual and theoretical ethanol yields for switchgrass strains using NIRS analyses. BioEnergy Res.

[14] Hou S, Li L (2011). Rapid characterization of woody biomass digestibility and chemical composition using near-infrared spectroscopy. J Integr Plant Biol.

[15] Lorenzana RE, Lewis MF, Jung H-JG, Bernardo R (2010). Quantitative trait loci and trait correlations for maize stover cell wall composition and glucose release for cellulosic ethanol. Crop Sci.

[16] Hames B, Kruse T, Thomas SR, Ragab AS (2013). Method for predicting the amount of accessible carbohydrate in a feedstock sample using a near-infrared model. US Patent.

[17] Adler PR, Sanderson MA, Weimer PJ, Vogel KP. Plant Species Composition and Biofuel Yields of Conservation Grasslands. 2009, 19:2202–2209 10.1890/07-2094.1 20014588

[18] Monono EM, Haagenson DM, Pryor SW (2012). Developing and evaluating NIR calibration models for multi-species herbaceous perennials. Ind Biotechnol.

[19] Da Silva PD, Guillemain A, Laballette F (2011). Characterisation of feedstock biorefinery raw material by near infrared spectroscopy. 16th International Symposium on Wood, Fiber, and Pulping Chemistry, V.

[20] Liu L, Ye XP, Womac AR, Sokhansanj S (2010). Variability of biomass chemical composition and rapid analysis using FT-NIR techniques. Carbohydr Polym.

[21] Hodge G, Woodbridge W (2010). Global near infrared models to predict lignin and cellulose content of pine wood. J Near Infrared Spectrosc.

[22] Chataigner F, Surault F, Huyghe C and Julier B. Determination of Botanical Composition in Multispecies Forage Mixtures by Near Infrared Reflectance Spectroscopy. In Sustainable Use of Genetic Diversity in Forage and Turf Breeding. Edited by Huyghe C. Springer; 2010:199–203.

[23] Mika V, Pozdisek J, Tillmann P, Nerusil P, Buchgraber K, Gruber L (2003). Development of NIR calibration valid for two different grass sample collections. Czech J Anim Sci.

[24] Sanderson MA, Agblevor F, Collins M, Johnson DK (1996). Compositional analysis of biomass feedstocks by near infrared reflectance spectroscopy. Biomass Bioenergy.

[25] Dale LM, Thewis A, Rotar I, Boudry C, Pacurar FS, Lecler B (2013). Fertilization effects on the chemical composition and *in vitro* organic matter digestibility of semi-natural meadows as predicted by NIR spectrometry. Not Bot Horti Agrobot Cluj-Napoca.

[26] Dien BS (2010). Mass Balances and Analytical Methods for Biomass Pretreatment Experiments. Biomass to Biofuels: Strategies for Global Industries.

[27] Per Å. Composition and Structure of Cell Wall Polysaccharides in Forages. In Forage Cell Wall Structure and Digestibility. Volume Acsesspubl. Edited by Jung HG, Buxton DR, Hatfield RD, and Ralph J. American Society of Agronomy, Crop Science Society of America, Soil Science Society of America; 1993:183–199.

[28] Jung H-JG, Valdez FR, Hatfield RD, Blanchette RA (1992). Cell wall composition and degradability of forage stems following chemical and biological delignification. J Sci Food Agric.

[29] Thammasouk K, Tandjo D, Penner MH (1997). Influence of extractives on the analysis of herbaceous biomass. J Agric Food Chem.

[30] Johnson DK, Ashley PA, Deutch SP, Davis MF, Fennell JA, Wiselogel A (1995). Compositional Variability in Herbaceous Energy Crops. Second Biomass Conference of the Americas: Energy, Environment, Agriculture, and Industry Proceedings.

[31] Wolfrum EJ, Ness RM, Nagle NJ, Peterson DJ, Scarlata CJ (2013). A laboratory-scale pretreatment and hydrolysis assay for determination of reactivity in cellulosic biomass feedstocks. Biotechnol Biofuels.

[32] Templeton DW, Scarlata CJ, Sluiter JB, Wolfrum EJ (2010). Compositional analysis of lignocellulosic feedstocks. 2. Method uncertainties. J Agric Food Chem.

[33] Wolfrum E, Payne C, Stefaniak T, Rooney W, Dighe N, Bean B (2013). Multivariate Calibration Models for Sorghum Composition Using Near-Infrared Spectroscopy, Technical Report NREL/TP-510056838.

[34] Haffner FB, Mitchell VD, Arundale RA, Bauer S (2013). Compositional analysis of Miscanthus giganteus by near infrared spectroscopy. Cellulose.

[35] Hattori T, Murakami S, Mukai M, Yamada T, Hirochika H, Ike M (2012). Rapid analysis of transgenic rice straw using near-infrared spectroscopy. Plant Biotechnol.

[36] Xu F, Zhou L, Zhang K, Yu J, Wang D: Rapid Determination of Both Structural Polysaccharides and Soluble Sugars in Sorghum Biomass Using Near-Infrared Spectroscopy. BioEnergy Res 2014:1–7

[37] Guimarães CC, Simeone MLF, Parrella RAC, Sena MM (2014). Use of NIRS to predict composition and bioethanol yield from cell wall structural components of sweet sorghum biomass. Microchem J.

[38] Lomborg CJ, Thomsen MH, Jensen ES, Esbensen KH (2010). Power plant intake quantification of wheat straw composition for 2nd generation bioethanol optimization—a near infrared spectroscopy (NIRS) feasibility study. Bioresour Technol.

[39] Standard Procedures for Biomass Compositional Analysis [http://www.nrel.gov/biomass/analytical_procedures.html]

[40] Kennard RW, Stone LA (1969). Computer aided design of experiments. Technometrics.

[41] Sluiter JB, Ruiz RO, Scarlata CJ, Sluiter AD, Templeton DW (2010). Compositional analysis of lignocellulosic feedstocks. 1. Review and description of methods. J Agric Food Chem.

[42] R: a language and environment for statistical computing [http://www.r-project.org/]

